# Epidemiological aspects of the persistent transmission of rabies during an outbreak (2010 – 2017) in Harare, Zimbabwe

**DOI:** 10.1371/journal.pone.0210018

**Published:** 2019-01-10

**Authors:** Andre Coetzer, Lambert Gwenhure, Pious Makaya, Wanda Markotter, Louis Nel

**Affiliations:** 1 Department of Biochemistry, Genetics and Microbiology, Faculty of Natural and Agricultural Sciences, University of Pretoria, Pretoria, Gauteng, South Africa; 2 Global Alliance for Rabies Control SA NPC, Pretoria, Gauteng, South Africa; 3 Department of Livestock & Veterinary Services, Central Veterinary Research and Diagnostic Laboratory, Harare, Harare metropolitan province, Zimbabwe; 4 Department of Medical Virology, Centre for Viral Zoonoses, University of Pretoria, Pretoria, Gauteng, South Africa; Faculty of Science, Ain Shams University (ASU), EGYPT

## Abstract

Canine-mediated human rabies is endemic to the entire African continent, where the disease burden is often highest in rural communities of resource-limited countries. In this study, we analysed an animal rabies outbreak, which had persisted since 2010 in the predominantly metropolitan capital city of Zimbabwe, Harare. As rabies is considered to disproportionally affect rural communities, the persistence of urban rabies in this metropolitan setting is of interest. In order to gain an improved understanding of the epidemiology of the outbreak under investigation, we utilised both routine surveillance data that had been collected during the first eight years of the outbreak (2010–2017), as well as molecular epidemiological analyses relying on the Bayesian Markov Chain Monte Carlo methodology. This approach allowed us to characterize virus transmission by identifying specific suburbs within the city limits where persistent disease transmission took place, while also confirming that immunologically naïve dogs were the most likely reservoir species in and around the city. In addition to gaining an improved local understanding of the outbreak, we are also able to infer that rabies was likely introduced to the city in 2010 when a rabid animal was moved from the north-east of Zimbabwe into Harare–resulting in an epizootic event. The work presented here not only showcased the value of combining conventional and molecular epidemiological data, but also highlighted the importance of maintaining rabies vaccination coverage and continued public awareness in urban areas where the risk appears to be low. By educating the general population on rabies and relying on owners to bring their companion animals to strategically placed vaccination points, the control and elimination of rabies from Harare may be feasible.

## Introduction

Rabies, caused primarily by the *Rabies lyssavirus* (RABV), is a neglected zoonotic disease that is transmitted mainly by domestic dogs (*Canis lupus familiaris*), significantly affecting humans in developing countries on the Asian and African continents [1]. Although canine-mediated rabies has been successfully controlled and eliminated by some countries [1–4], predictive burden models estimate that the disease still kills more than 59,000 people every year, with twenty one thousand (36%) of those estimated deaths occur on the African continent alone [1,5].

The countries where canine-mediated human rabies had been eliminated showcased the importance of four independent workstreams, *viz*. i) enhanced disease surveillance and diagnostic confirmation, ii) improved access to both human and animal vaccines, iii) responsible dog ownership, and iv) enhanced education and awareness [2–4,6]. Of the various workstreams, controlling the disease in the dog population through vaccination has been shown to be the most important factor associated with minimising the risk to the public sector [1,2,7,8]. Unfortunately, human rabies cases still occur due to various factors that influence the effectiveness of the workstreams, thus contributing to the inadequate control of dog-mediated rabies in endemic areas [1,5,9]. Indeed, the situation is no different in the rabies-endemic country of Zimbabwe where an estimated 410 humans succumb to the entirely preventable disease annually [5,10].

The first recorded instance of rabies in Zimbabwe was reported in 1902 after an outbreak of the disease in the neighbouring country of Zambia [11–13]. The outbreak of rabies rapidly spread throughout Zimbabwe but was brought under control in 1913 after strict dog management measures had been implemented [13,14]. Subsequently, Zimbabwe was believed to be free of canine-mediated rabies for more than 30 years (1913–1950) with only two dogs presenting with signs of rabies while being subjected to mandatory quarantine at the border during that time [15,16]. In the 1950s, rabies was reintroduced to Zimbabwe from either Botswana or South Africa [11]. After the second introduction, rabies became established throughout Zimbabwe in both the domestic dog populations and terrestrial sylvatic carnivores (primarily side-striped jackal (*Canis adustus*) and black-backed jackals (*Canis mesomelas*)) [17–22].

While sylvatic reservoir species are generally limited to Zimbabwe’s commercial farming areas, where they cause comparatively few human exposures, canine-mediated rabies is primarily maintained in the densely populated rural settlements. Previous studies have shown that approximately 70% of Zimbabwe’s dog population is found in these resource-limited settlements, putting the cohabiting communities at high risk of canine-mediated human rabies [17,23,24]. In order to infer disease spread and identify endemic cycles, the transmission dynamics of canine-mediated rabies was further defined with the use of molecular epidemiological analyses in 2003, which found that independent endemic cycles of rabies–defined by species, location and time–existed within Zimbabwe [17]. In contrast to the rural areas of Zimbabwe, the more developed urban areas were historically not associated with significant rabies outbreaks. In support of this finding, historical data collected over a 13-year period (1985 to 1996) revealed that 56% of rabies-positive cases originated in the country’s rural settlements, 31% in commercial farming areas and only 13% in urban areas [19].

Due to the public health risk that canine-mediated rabies poses on the human population of Zimbabwe, mass dog vaccination (MDV) campaigns were implemented with varying degrees of success from 1951 onwards. A marked decline in the vaccination coverage was noted from 1990 onwards when resources were reallocated to other disease outbreaks within the country [15,19]. The limited resources allocated towards the control of rabies in Zimbabwe restricted disease interventions to annual static-point vaccination campaigns that focussed primarily on high-risk dog populations in the country’s rural settlements. As such, the urban areas were often neglected during these campaigns, and the estimated vaccination coverage in cities and larger towns declined systematically [15,19].

The neglect of the urban areas during annual MDV campaigns became evident in 2010 when a noteworthy number of rabies-positive cases were detected in the capital city of Harare. The city of Harare, which is situated within the slightly larger metropolitan Harare province, is considered the most densely populated province in Zimbabwe with 99% of the province’s population residing in an urban setting [25]. A few cases of animal rabies were detected in areas surrounding the city of Harare in the years leading up to the outbreak (four cases in 2008 and three cases in 2009). Despite a high human and animal density, no rabies cases were detected inside the city. However, rabies-positive cases emerge and continued to increase inside the city limits from 2010 onwards. Eventually, the government officially declared an outbreak of rabies in the animal population of the city of Harare in 2016 [26]. While the duration of the rabies in Harare could be indicative of an enzootic event, the anecdotal evidence suggested that rabies had been endemic to Zimbabwe for more than 60 years [11]. As such, the duration of the rabies outbreak in Harare had not only been comparatively short, but also maintained within a relatively defined geographical area–resulting in it being defined as an epizootic event [27].

Apart from identifying the outbreak through active surveillance, no additional epidemiological analyses were undertaken and the transmission dynamics of the outbreak remained poorly defined. Our aim was to contribute to a better understanding of the epidemiology of animal rabies in Harare. In order to do so, we analysed both surveillance and molecular epidemiological data pertaining to the ongoing rabies outbreak. We collated and analysed routine surveillance data from the first eight years of the outbreak (2010–2017) and also conducted a molecular epidemiological analysis of samples collected during the outbreak using the Bayesian Markov Chain Monte Carlo method. This approach enabled us to investigate the genetic relatedness of viruses collected from Harare to those historically collected from elsewhere in Zimbabwe as well as the neighbouring countries of Mozambique and Zambia in an effort to improve the resolution of the surveillance data.

## Materials and methods

### Ethics statement

Rabies is a notifiable disease in Zimbabwe and all animal health professionals were thus mandated to undertake routine animal rabies surveillance throughout the country. While owner consent was requested at all times, animal health professionals were authorised by law to humanely euthanize and collect samples from any suspect animals for rabies diagnosis through Zimbabwe’s public health act (Chapter 15:09) [28].

Furthermore, the Animal Ethics Committee (AEC) at the University of Pretoria (South Africa) provided ethical approval for the molecular epidemiological work described here (Approval numbers: EC027-16).

### Sample collection and diagnosis

Once specimens had been collected from suspect rabid animals, the brain samples were sent to the central veterinary laboratory (CVL) in Harare where routine rabies diagnosis was undertaken by means of both the direct fluorescent antibody (DFA) [29,30] and the direct, rapid immunohistochemical test (DRIT) [31] assays.

### Analysis of epidemiological surveillance data collected during the outbreak

Data on all of the rabies-positive and -negative samples collected from in and around the Harare city limits between 2010 and 2017 were collated and defined according to the following parameters: i) date, ii) animal species, iii) ownership information, v) suburb and provincial region, and vi) either the exact or approximated spatial coordinates. The spatiotemporal geographical plotting of the rabies-positive cases was visualized using the Tableau software package (version 10.5) [32].

### Molecular epidemiology of rabies in the capital city of Zimbabwe

#### Viral RNA extraction and reverse transcription

Twenty-three rabies-positive brain samples were chosen based on their location within the city and the available brain material stored at the CVL. This representative cohort of brain samples, collected from each of Harare’s five metropolitan regions over a three-year period (2014–2016), was used for molecular characterization and phylogenetic analysis. The total RNA was extracted using the Direct-zol RNA MiniPrep Plus kit (Zymo Research) before viral cDNA synthesis was performed [33].

#### Polymerase chain reaction and sequencing

The cytoplasmic domain of the glycoprotein gene and the adjacent G-L intergenic region of the RABV genome was subsequently amplified using the G(+) and L(-) primer sets previously published [34,35]. The amplified PCR-positive products (*n = 23*) were electrophoresed and visualized on a standard 1% agarose gel before being purified using the Zymoclean Gel DNA Recovery kit according to the manufacturer’s instructions (Zymo Research).

Both the forward and reverse strands of the purified PCR amplicons were subjected to Sanger sequencing using the respective PCR primers and the BigDye Terminator v3.1 sequencing reaction kit (Applied Biosystems). The sequencing was undertaken on a ABI3500XL automated capillary sequencer (Inqaba Biotec, RSA) and the final consensus sequences were trimmed to 592 nucleotides (nt) using the CLC Main Workbench software (CLC Bio, Version 7.7.2) [36]. The consensus sequences were allocated unique accession numbers (MF425791—MF425813) and submitted to the NCBI GenBank.

#### Phylogenetic analysis

While the phylogenetic analysis of a single region of the virus genome has limitations [37], the G-L intergenic region has been shown to be particularly useful in various previous investigations. Some of these studies also demonstrated that basic phylogenetic interpretations from analyses relying on either the complete G protein gene or the adjacent G-L intergenic region were equivalent for RABV [22,35,38–40]. This is also the case for rabies-related lyssaviruses where the fundamental phylogenetic patterns observed with the use of either the N or the G protein genes were shown to be equivalent [41].

In our study, a preliminary phylogenetic analysis included G-L intergenic region sequences from Zimbabwe as well as the neighbouring countries of Mozambique, Namibia, South Africa and Zambia. However, the RABV sequences from Namibia and South Africa did not contribute towards a better interpretation of the rabies outbreak in Harare and were omitted from the final phylogenetic analysis reported here. The final phylogenetic analysis thus included a total of 80 G-L intergenic region sequences obtained from Zimbabwe and the neighbouring countries of Mozambique and Zambia (S1 Table).

An alignment of the 80 sequences was created using the ClustalW subroutine of the BioEdit software [42] and the best fitting DNA substitution model (TIM1+G) was determined using the Akaike’s information criterion (AIC) within the JModel software package (version 2.1.3) [43]. The phylogenetic analysis was undertaken with the BEAST software package (version 1.8.1) [44] using a Bayesian Markov Chain Monte Carlo (MCMC) method. The phylogenetic analysis relied on three independent Markov chains that were sampled for 10 million states. A sampling frequency of 10,000 was combined after discarding at least 10 percent burn and inspecting the posterior distributions to ensure adequate mixing and convergence. A maximum clade credibility tree was subsequently compiled to summarize the associated statistics and the FigTree software package (version 1.4.2) [45] was used to visualize the phylogenetic tree.

## Results and discussion

The work presented here exemplified the use of surveillance and molecular epidemiological data to create an improved understanding of a canine-mediated rabies outbreak in a metropolitan African setting. To date, two previous studies focusing on rabies outbreaks in urban areas endeavoured to gain an improved understanding of the disease dynamics using either surveillance or molecular epidemiological data [46,47]. Furthermore, only one other study on the African continent had used a methodology similar to ours, i.e. an analysis of both routine surveillance and molecular epidemiological data towards characterizing the prevalence and endemicity of rabies cases within an urban setting [48].

### Epidemiological description of the rabies outbreak in the Harare province

**Fig 1:**
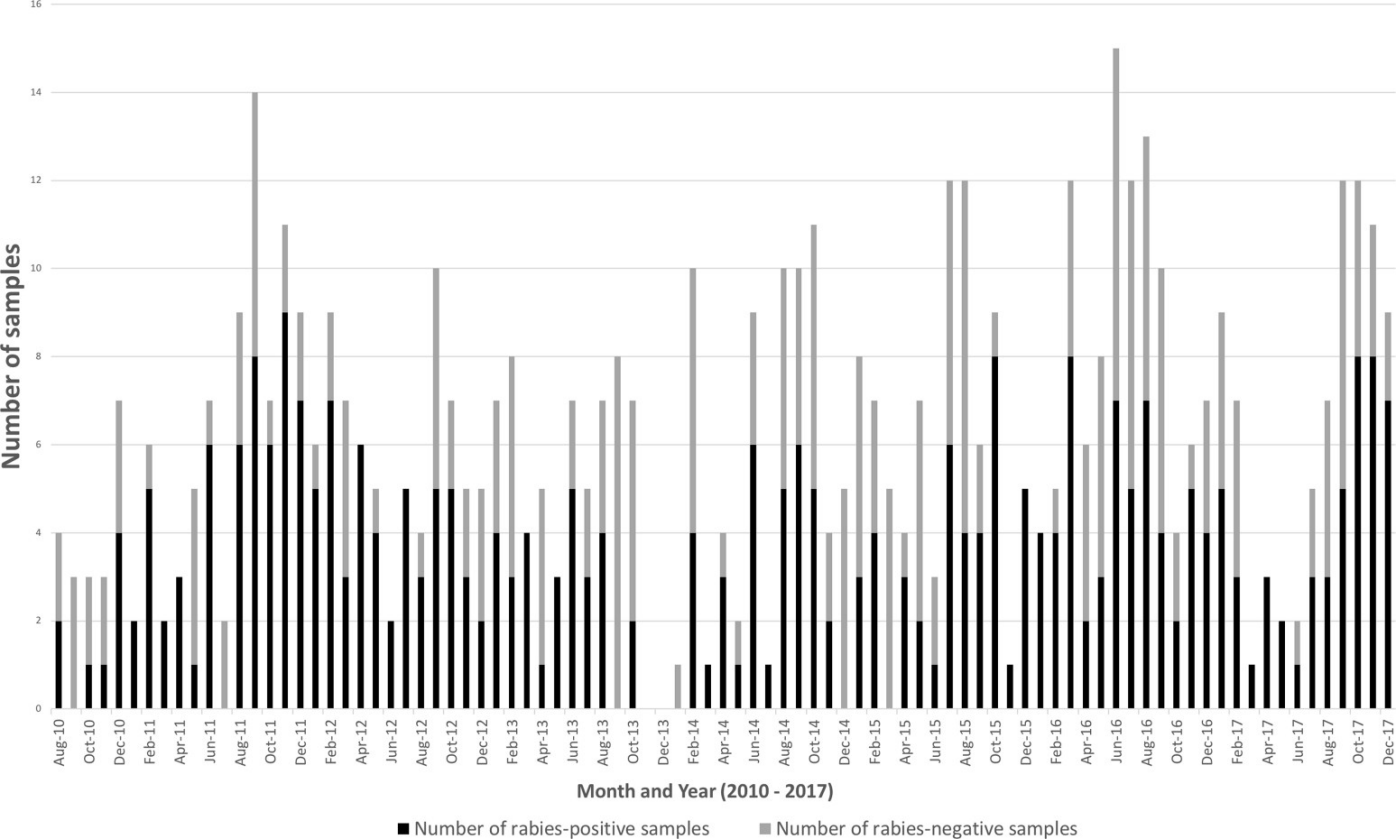
The number of rabies-positive and -negative samples collected per month from within and around the Harare Metropolitan Province between 2010 and 2017. Black bars represent the number of rabies-positive samples and grey bars represent the number of rabies-negative samples.

Between 2010 and 2017, samples from 552 animals were collected from Harare and subjected to rabies testing (S2 Table and Fig 1). Of these, 316 animal samples (57%) were rabies-positive (S2 Table and Fig 1). The lowest number of rabies-positive samples (*n = 8*) was in 2010 (with 20 suspect animal rabies samples tested that year). Since then, the numbers were consistently higher in subsequent years (average of 44 rabies-positive samples collected per year between 2011 and 2017) (S2 Table and Fig 1). While the detection of rabies-negative cases from all of the city’s regions was indicative of the robustness of the rabies surveillance network in Harare, the persistently high number of rabies-positive cases detected throughout the outbreak signified that disease transmission was most likely occurring in the city.

**Fig 2:**
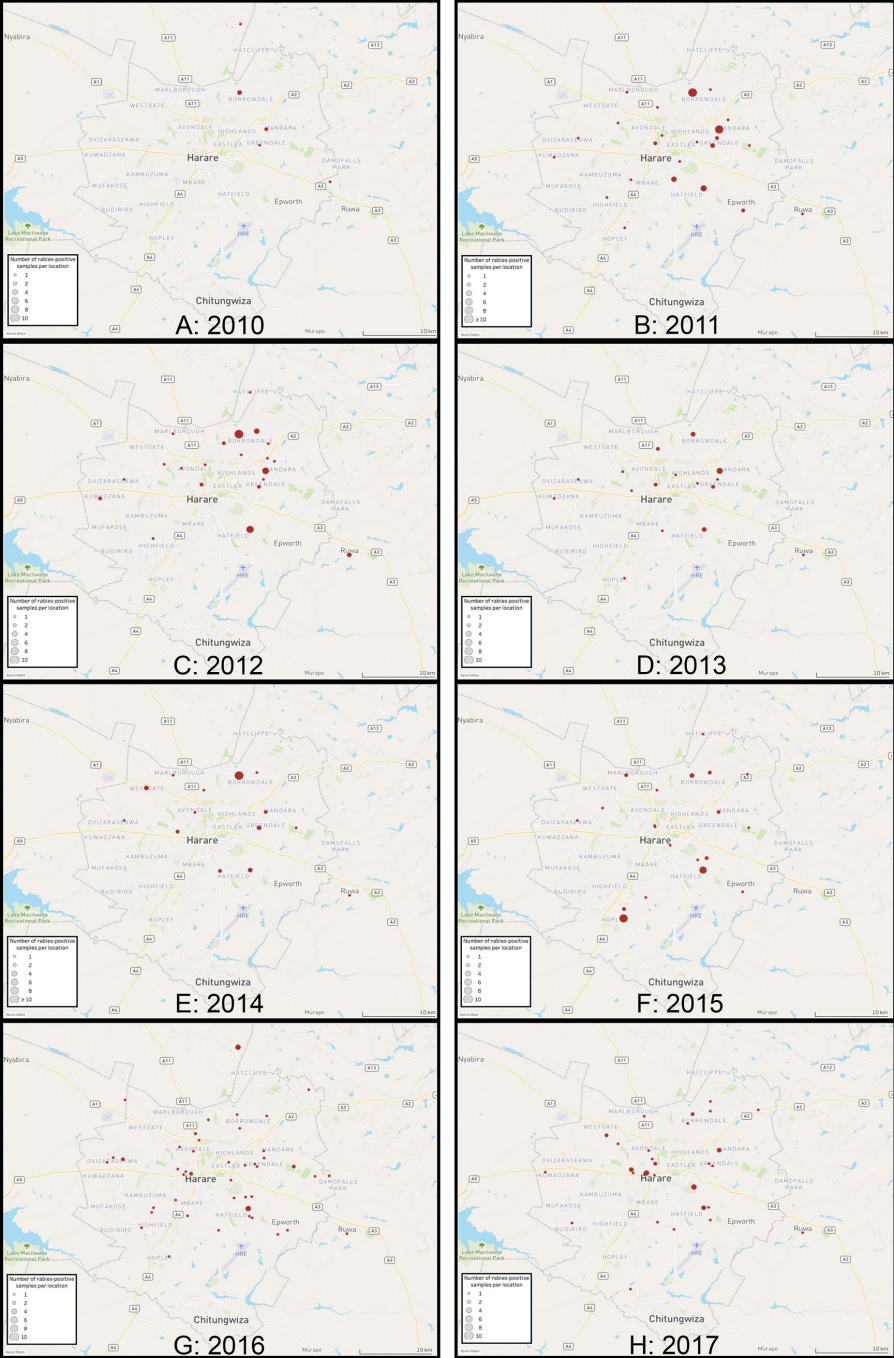
Spatio-temporal geographical maps of the Harare city limits showing the location of all the rabies-positive (red dots) samples detected between 2010 and 2017. Each map represents the surveillance data collected over the calendar year for the following times: A) 2010, B) 2011, C) 2012, D) 2013, E) 2014, F) 2015, G) 2016 and H) 2017.

Furthermore, spatio-temporal geographical mapping of animal rabies cases revealed that rabies was detected in the North-eastern suburbs of the city limits for the first time in 2010, from where rabies appeared to have spread unpredictably throughout the city’s animal populations (Fig 2). In support of this finding, animal rabies cases were detected in 50/137 (36%) of Harare’s suburbs between 2010 and 2017 (S3 Table). Although animal rabies cases appeared widely distributed throughout the city over the eight-year period, spatio-temporal analysis of the rabies-positive samples identified persistent disease transmission amongst animals in the North-eastern (136/316 (43%)) and Southern (63/316 (20%)) regions of the city (S3 Table and Fig 2).

By disaggregating the animal samples according to their species of origin, we found that 264 (84%) of all rabies-positive samples came from domestic dogs from within and around the metropolitan area, suggesting that virus transmission was predominantly maintained within the immunologically naive canine population residing within the city limits. From 2014 onwards, additional epidemiological information on the ownership of all suspect rabid dogs was collected. This variable indicated that 124 of the 155 (80%) rabies-positive dogs tested between 2014 and 2017 were “owned dogs” with identified owners (S2 Table). The remaining rabies-positive animal samples collected had originated from either wildlife species residing around Harare (black-backed jackal, *n = 11*; duiker, *n = 1*, civet, *n = 1*; lion, *n = 1*; zebra, *n = 1*; and kudu, *n = 1*), or mammalian species found within the metropolitan area (bovine, *n = 18*, feline, *n = 15*; porcine, *n = 1*; equine, *n = 1;* and an unidentified rodent species, *n = 1*) (S2 Table).

### Molecular epidemiology

The phylogenetic analysis undertaken in this study illustrated that all of the sequences included in the molecular epidemiological analysis, including the 23 new RABV sequences collected from Harare, formed part of the Cosmopolitan clade of RABV–a clade which is believed to have originated in the Palearctic region before spreading through most of the African continent [37,49,50]. More specifically, the sequences were found to group within the Africa 1b sub-lineage that is predominantly found in South-east Africa [37,51]. This observation was, however, not surprising as previous investigations found the canine-variant of RABV in southern and eastern Africa to be genetically distinct from those circulating in the western and central regions of the continent [13,49,51–53].

**Fig 3:**
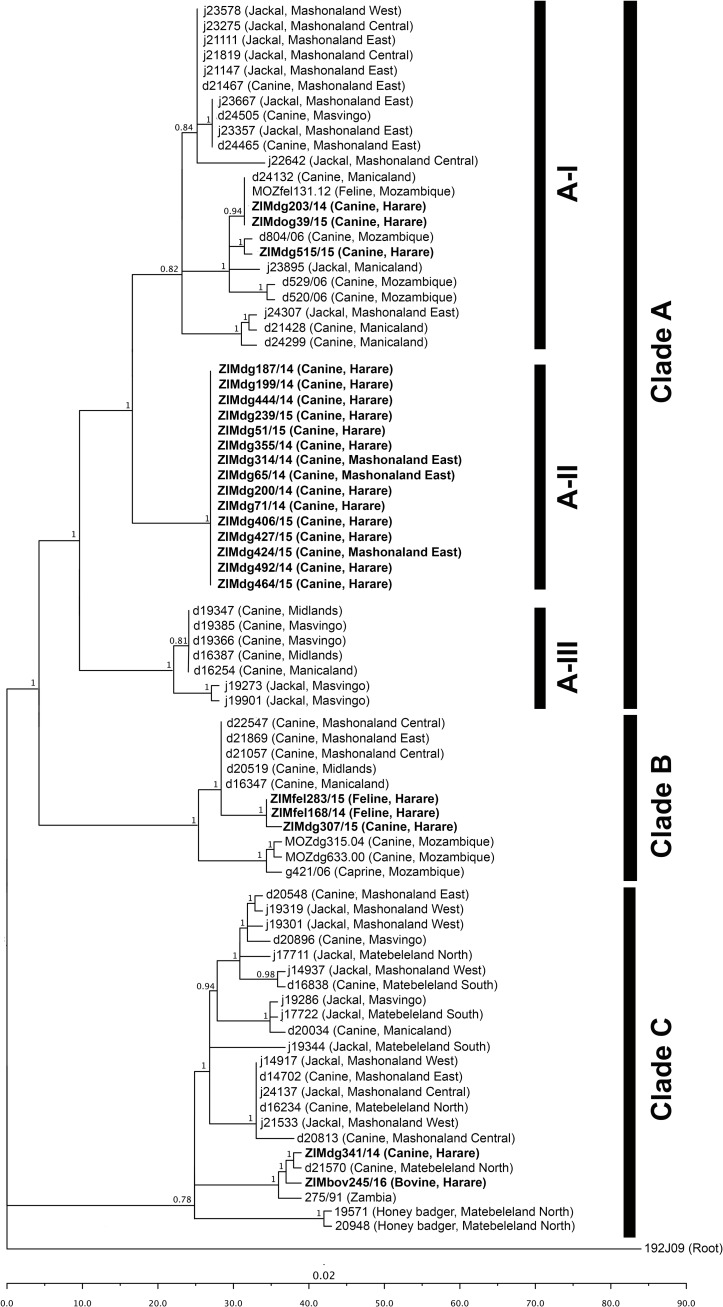
Maximum clade credibility tree of the cytoplasmic domain of the G-L intergenic region of RABV sequences originating from Zimbabwe and selected neighbouring countries (Zambia and Mozambique). The scale bar depicts nucleotide substitutions per site (mean: 2,4E-4 substitutions/site) and all branches with a posterior probability of 0.75 or less were collapsed. A sequence from a jackal rabies virus strain from Namibia (192J09) was used to root the tree. The new sequences generated in this study, all clustering within the Africa 1b sub-lineage of the Cosmopolitan clade of RABV, are indicated in a bold font.

At a more localized level, the RABV sequences included in this study could phylogenetically be divided into three clades (Clade A–C), with each clade being supported by posterior probability scores of 1, 1 and 0.78 respectively (Fig 3). Clade A could be further divided into 3 separate sub-clades (Sub-clade AI–AIII) with each sub-clade representing independent endemic cycles that were geographically defined, despite being genetically related (Fig 3).

Sub-clade A-I consisted of RABV sequences that had been obtained primarily from the northern and eastern regions of Zimbabwe. The branch clustering observed in this sub-clade was similar to those reported in an investigation of the molecular epidemiology of canine-mediated rabies in Zimbabwe [17]. Our results, however, provided evidence that RABV-positive samples from the Harare province of Zimbabwe (*n = 3*) and Mozambican provinces of Manica (*n = 3*) and Nampula (*n = 1*) were also part of this specific endemic cycle (Fig 3). Sub-clade A-II, consisted exclusively of RABV sequences (*n = 15*) that had been collected from in and around the Harare city limits (Fig 3). The viruses within this cluster are genetically homologous and probably represent a clonal population that is phylogenetically most closely related to the populations represented in Sub-clade A-I. The third sub-clade, Sub-clade A-III, consisted of RABV sequences (*n = 7*) obtained from the southern region of Zimbabwe. This branch clustering was consistent with those reported during the previous investigation of the molecular epidemiology of canine-mediated rabies in the country [17] (Fig 3). The grouping of sequences represented by Clade A suggested that the viruses cycling within the city limits were genetically related to those from an endemic cycle that ranged from north-eastern Zimbabwe to the neighbouring country of Mozambique (Fig 3). This finding supported our initial hypothesis that the introduction of rabies into the North-eastern region of Harare city had resulted in sustained disease transmission in the urban area. In further support, a previous investigation of a rabies outbreak in an urban area in South Africa had also found that a single introduction of a rabid animal could result in a widespread epizootic event [47].

Clade B primarily consisted of rabies-positive samples (*n = 11*) that, like those in Sub-clade A-I, had been collected from the north-eastern region of Zimbabwe (Fig 3). These samples were, however, phylogenetically distinct from those in Sub-clade A-I (Fig 3). While similar result had been reported in a previous investigation [17], our results provide evidence that RABV-positive samples identified in Harare (*n = 3*) and Mozambique (*n = 3*) could also be linked to this endemic cycle (Fig 3). Clade C comprised of rabies-positive samples (*n = 23*) collected primarily from the central region of Zimbabwe (Fig 3). While the branch clustering observed in this clade is similar to findings seen in an earlier study [17], our results provide evidence that RABV-positive samples identified in the capital city of Harare (*n = 2*) and Zambia (*n = 1*) are also linked to this endemic cycle.

The fact that five of the rabies-positive cases collected within the Harare city limits during the outbreak did not form part of either the endemic cycle in the capital city (Sub-clade A-II) or the cycle found in north-eastern Zimbabwe (Sub-clade A-I) indicated that rabies had been introduced into the capital city of Harare on more than one occasion (Fig 3). Indeed, the five samples (ZIMfel283/15; ZIMfel168/14; ZIMdg307/15; ZIMdg341/14 and ZIMbov245/16) were phylogenetically related to either one of two distinct and distant endemic cycles, one of which persisted in the north-eastern (Clade B) and the other in the central region of Zimbabwe (Clade C) [17] (Fig 3).

The observed genetic homogeneity among viruses collected from a single geographical location could be explained by the movement of humans and their companion animals [37,40]. By moving from rural to urban and peri-urban areas, dog owners may inadvertently facilitate the transmission of rabies over long distances [37,40]. Such movement into areas that are populated by predominantly immunologically naïve animals–such as Harare city–could explain the establishment of new endemic cycles of rabies that places an even greater burden on the already resource-limited infrastructure.

## Conclusion

The work presented in this study highlighted the importance of the four independent workstreams that play a role in the control and elimination of canine-mediated rabies [6]. In our investigation, the collection, collation and analysis of surveillance and molecular epidemiological data provided insight into how the disease most likely entered the city, as well as evidence of the sustained, long-term transmission of rabies in the city’s dog population. While these findings helped us to gain an improved understanding of the epidemiology of animal rabies in the Harare city limits, they also stressed the need for a specific disease-intervention campaign in order to control the ongoing epizootic outbreak. Given the desperately limited resources available to the country, the control and elimination of the animal rabies outbreak could follow a strategy that starts with the vaccination of the most at-risk dog populations (e.g. based on number of cases and transmission rates), such as those residing in the North-eastern and Southern regions of the city. By prioritizing the implementation of vaccination campaigns to those specific suburbs, the disease transmission could be interrupted in a relatively short period, allowing the campaign to then progress in a wave-like manner until at least 70% of the city’s dog population has been vaccinated [1].

While the implementation of dog vaccination campaigns in response to the ongoing outbreak within the city limits should be emphasized, our findings also highlight the crucial role that the responsible dog ownership and enhanced public awareness workstreams play in the elimination of rabies [6]. Eighty percent of the rabies-positive dogs identified in the city between 2014 and 2017 were considered “owned” animals that should have been vaccinated according to existing legislation [54]. Awareness campaigns educating the public on rabies and the importance of complying with existing legislation pertaining to rabies vaccination campaigns could therefore contribute significantly to controlling the outbreak by ensuring that dog owners take responsibility for the vaccination of their animals. This approach would ensure the vaccination coverage of a large proportion of the dog population, while also reducing reliance on the costly and time-consuming trap-vaccinate-release (TVR) method of vaccinating free-roaming dogs.

In summary, the results of this study not only improved our understanding of the rabies outbreak under investigation, but also emphasized the importance of the implementation of both strategic vaccination campaigns and public awareness campaigns focusing on responsible dog ownership in an effort to control the epizootic outbreak. Without implementing the aforementioned disease intervention initiatives, rabies runs the risk of becoming enzootic to the capital city of Zimbabwe, complicating the sustainable control and elimination of the disease.

## Supporting information

S1 Table A panel of rabies virus partial DNA sequences from Zimbabwe and neighbouring countries included in the phylogenetic analysis performed in this study. (PDF)

S2 Table Epidemiological surveillance data collected between 2010 and 2017 in and around the Harare city limits during an outbreak of canine-mediated rabies. (PDF)

S3 Table Distribution of rabies-positive samples within the suburbs and peri-urban areas of the Harare Metropolitan province during the outbreak, 2010–2017. (PDF)
