# Supplementary material for: Epidemiological aspects of the persistent transmission of rabies during an outbreak (2010 – 2017) in Harare, Zimbabwe
Source: PLoS One. 2019 Jan 10;14(1):e0210018. doi: 10.1371/journal.pone.0210018 (PMC6328171; doi:10.1371/journal.pone.0210018)
Supplement: S1 Table — (PDF) [file pone.0210018.s001.pdf]

S1. Table. A panel of rabies virus partial DNA sequences from Zimbabwe and neighbouring countries included in the phylogenetic analysis performed in this study.

Number	Sample Number	Species	Country	Province	Latitude	Longitude	Accession number
1	MOZdog633/00	Canine	Mozambique	Nampula	-15,1266347	39,2687161	KM262040
2	MOZdog315/04	Canine	Mozambique	Maputo	-25,969248	32.,731746	KM262041
3	MOZfel131/12	Feline	Mozambique	Manica	-19.5059787	33,438353	KM262046
4	g421/06	Caprine	Mozambique	Cabo Delgado	-19.5059787	33,438353	EU123930
5	d520/06	Canine	Mozambique	Nampula	-15,1266347	39,2687161	EU123931
6	d529/06	Canine	Mozambique	Manica	-19.5059787	33,438353	EU123932
7	d804/06	Canine	Mozambique	Manica	-19.5059787	33,438353	EU123934
8	275/91	Canine	Zambia	Unkown	-15,3875259	28,3228165	GQ983449
9	20948	Honey badger	Zimbabwe	Matabeleland North	-18,5331566	27,5495846	AF3041891
10	19571	Honey badger	Zimbabwe	Matabeleland North	-18,5331566	27,5495846	AF3041841
11	j19319	Jackal	Zimbabwe	Mashonaland West	-17,4851029	29,7889248	AF177082
12	d20548	Canine	Zimbabwe	Mashonaland East	-18,5871642	31,2626365	AF177061
13	j19301	Jackal	Zimbabwe	Mashonaland West	-17,4851029	29,7889248	AF177081
14	d20896	Canine	Zimbabwe	Masvingo	-20,6241509	31,2626365	AF177062
15	d16838	Canine	Zimbabwe	Matabeleland South	-21,052337	29,0459926	AF177058

16	j14937	Jackal	Zimbabwe	Mashonaland West	-17,4851029	29,7889248	AF177077
17	j17711	Jackal	Zimbabwe	Matabeleland North	-18,5331566	27,5495846	AF177078
18	j19344	Jackal	Zimbabwe	Matabeleland South	-21,052337	29,0459926	AF303077
19	d14702	Canine	Zimbabwe	Mashonaland East	-18,5871642	31,2626365	AF177053
20	d20813	Canine	Zimbabwe	Mashonaland Central	--16,7644294	31,0793704	AF177063
21	j14917	Jackal	Zimbabwe	Mashonaland West	-17,4851029	29,7889248	AF177076
22	d16234	Canine	Zimbabwe	Matabeleland North	-18,5331566	27,5495846	AF177054
23	j21533	Jackal	Zimbabwe	Mashonaland West	-17,4851029	29,7889248	AF177085
24	j24137	Jackal	Zimbabwe	Mashonaland Central	--16,7644294	31,0793704	AF177095
25	j19286	Jackal	Zimbabwe	Masvingo	-20,6241509	31,2626365	AF177080
26	d20034	Canine	Zimbabwe	Manicaland	-18,9216386	32,1746049	AF177059
27	j17722	Jackal	Zimbabwe	Matabeleland South	-21,052337	29,0459926	AF177079
28	d24465	Canine	Zimbabwe	Mashonaland East	-18,5871642	31,2626365	AF177074
29	j23667	Jackal	Zimbabwe	Mashonaland East	-18,5871642	31,2626365	AF177093
30	j23357	Jackal	Zimbabwe	Mashonaland East	-18,5871642	31,2626365	AF177090
31	j22642	Jackal	Zimbabwe	Mashonaland Central	--16,7644294	31,0793704	AF177088
32	j21147	Jackal	Zimbabwe	Mashonaland East	-18,5871642	31,2626365	AF177084
33	j21819	Jackal	Zimbabwe	Mashonaland Central	--16,7644294	31,0793704	AF177087
34	j21111	Jackal	Zimbabwe	Mashonaland East	-18,5871642	31,2626365	AF177083

35	j23578	Jackal	Zimbabwe	Mashonaland West	-17,4851029	29,7889248	AF177092
36	j24307	Jackal	Zimbabwe	Mashonaland East	-18,5871642	31,2626365	AF177096
37	d21467	Canine	Zimbabwe	Mashonaland East	-18,5871642	31,2626365	AF177066
38	j23275	Jackal	Zimbabwe	Mashonaland Central	--16,7644294	31,0793704	AF177089
39	d21428	Canine	Zimbabwe	Manicaland	-18,9216386	32,1746049	AF177065
40	d24299	Canine	Zimbabwe	Manicaland	-18,9216386	32,1746049	AF177073
41	j23895	Jackal	Zimbabwe	Manicaland	-18,9216386	32,1746049	AF177094
42	d24132	Canine	Zimbabwe	Manicaland	-18,9216386	32,1746049	AF177072
43	j19273	Jackal	Zimbabwe	Masvingo	-20,6241509	31,2626365	AF303076
44	j19901	Jackal	Zimbabwe	Masvingo	-20,6241509	31,2626365	AF303075
45	d19347	Canine	Zimbabwe	Midlands	--19,0552009	29,6035494	AF303079
46	d19385	Canine	Zimbabwe	Masvingo	-20,6241509	31,2626365	AF303080
47	d24505	Canine	Zimbabwe	Masvingo	-20,6241509	31,2626365	AF177075
48	d19366	Canine	Zimbabwe	Masvingo	-20,6241509	31,2626365	AF303078
49	d16387	Canine	Zimbabwe	Midlands	--19,0552009	29,6035494	AF177057
50	d16254	Canine	Zimbabwe	Manicaland	-18,9216386	32,1746049	AF177055
51	d22547	Canine	Zimbabwe	Mashonaland Central	--16,7644294	31,0793704	AF177070
52	d21057	Canine	Zimbabwe	Mashonaland Central	--16,7644294	31,0793704	AF177064
53	d20519	Canine	Zimbabwe	Midlands	--19,0552009	29,6035494	AF177060

54	d21869	Canine	Zimbabwe	Mashonaland East	-18,5871642	31,2626365	AF177069
55	d16347	Canine	Zimbabwe	Manicaland	-18,9216386	32,1746049	AF177056
56	d21570	Canine	Zimbabwe	Matabeleland North	-18,5331566	27,5495846	AF177068
57	**ZIMdg65/14**	Canine	Zimbabwe	Mashonaland East	-18,5871642	31,2626365	MF425791
58	**ZIMdg71/14**	Canine	Zimbabwe	Harare	- -17,8251657	31,0335099	MF425792
59	**ZIMfel168/14**	Feline	Zimbabwe	Harare	- -17,8251657	31,0335099	MF425793
60	**ZIMdg187/14**	Canine	Zimbabwe	Harare	- -17,8251657	31,0335099	MF425794
61	**ZIMdg199/14**	Canine	Zimbabwe	Harare	- -17,8251657	31,0335099	MF425795
62	**ZIMdg200/14**	Canine	Zimbabwe	Harare	- -17,8251657	31,0335099	MF425796
63	**ZIMdg203/14**	Canine	Zimbabwe	Harare	- -17,8251657	31,0335099	MF425797
64	**ZIMdg314/14**	Canine	Zimbabwe	Mashonaland East	-18,5871642	31,2626365	MF425798
65	**ZIMdg341/14**	Canine	Zimbabwe	Harare	- -17,8251657	31,0335099	MF425799
66	**ZIMdg355/14**	Canine	Zimbabwe	Harare	- -17,8251657	31,0335099	MF425800
67	**ZIMdg444/14**	Canine	Zimbabwe	Harare	- -17,8251657	31,0335099	MF425801
68	**ZIMdg492/14**	Canine	Zimbabwe	Harare	- -17,8251657	31,0335099	MF425802
69	**ZIMdg39/15**	Canine	Zimbabwe	Harare	- -17,8251657	31,0335099	MF425803
70	**ZIMdg51/15**	Canine	Zimbabwe	Harare	- -17,8251657	31,0335099	MF425804
71	**ZIMdg239/15**	Canine	Zimbabwe	Harare	- -17,8251657	31,0335099	MF425805
72	**ZIMfel283/15**	Feline	Zimbabwe	Harare	- -17,8251657	31,0335099	MF425806

73	**ZIMdg307/15**	Canine	Zimbabwe	Harare	- -17,8251657	31,0335099	MF425807
74	**ZIMdg406/15**	Canine	Zimbabwe	Harare	- -17,8251657	31,0335099	MF425808
75	**ZIMdg424/15**	Canine	Zimbabwe	Mashonaland East	-18,5871642	31,2626365	MF425809
76	**ZIMdg427/15**	Canine	Zimbabwe	Harare	- -17,8251657	31,0335099	MF425810
77	**ZIMdg464/15**	Canine	Zimbabwe	Harare	- -17,8251657	31,0335099	MF425811
78	**ZIMdg515/15**	Canine	Zimbabwe	Harare	- -17,8251657	31,0335099	MF425812
79	**ZIMbov245/16**	Bovine	Zimbabwe	Harare	- -17,8251657	31,0335099	MF425813
80	192J09 (Phylogenetic tree root)	Jackal	Namibia	Kunene	-19,4086317	13,914399	JX473839
Samples in bold text denotes the novel sequences generated in this study							
