# Supplementary material for: Epidemiological aspects of the persistent transmission of rabies during an outbreak (2010 – 2017) in Harare, Zimbabwe
Source: PLoS One. 2019 Jan 10;14(1):e0210018. doi: 10.1371/journal.pone.0210018 (PMC6328171; doi:10.1371/journal.pone.0210018)
Supplement: S2 Table — (PDF) [file pone.0210018.s002.pdf]

S2 Table. Epidemiological surveillance data collected between 2010 and 2017 in and around the Harare city limits during an outbreak of canine-mediated rabies.

Year	Animal species tested	Total number of samples tested for rabies	Total number of rabies-positive samples
2010	Canine	10	5
	Bovine	2	1
	Duiker	1	1
	Feline	3	1
	Rodent	3	0
	Ovine	1	0
2011	Canine	57	43
	Bovine	5	5
	Feline	6	2
	Equine	1	1
	Jackal	3	1
	Porcine	1	1
	Civet	1	0
	Rodent	1	0
2012	Canine	55	39
	Feline	4	4
	Bovine	4	3
	Jackal	3	2
	Civet	1	1
	Lion	1	1
	Ovine	1	0
	Porcine	1	0
	Rabbit	1	0
2013	Canine	47	22
	Feline	5	3
	Bovine	4	2
	Jackal	3	1
	Zebra	1	1
	Equine	1	0
2014	Canine	58	33<br>Owned: n = 15 (45%)
	Feline	5	1
	Rodent	1	0
	Jackal	1	0
	Bovine	1	0
	Wildebeest	1	0
	Bat	1	0
2015	Canine	64	35

			Owned: n = 29 (83%)
	Bovine	3	3
	Feline	5	1
	Kudu	2	1
	Rodent	1	1
	Equine	1	0
	Lion	1	0
	Monkey	1	0
	Zebra	1	0
2016	Canine	85	48<br>Owned: n = 48 (100%)
	Bovine	4	3
	Feline	4	1
	Jackal	1	1
	Bat	1	0
	Bushbaby	1	0
	Monkey	1	0
	Ovine	1	0
	Porcine	1	0
	Rabbit	1	0
2017	Canine	63	39<br>Owned: n = 32 (82%)
	Jackal	6	6
	Feline	4	2
	Bovine	3	1
	Genet	1	0
	Sable antelope	1	0

Canine: *Canis lupus familiaris*

Jackal: *Canis mesomelas*

Bovine: *Bos taurus*

Feline: *Felis catus*

Porcine: *Sus scrofa*

Equine: *Equus ferus caballus*

Duiker: *Philantomba monticola*

Civet: *Civettictis civetta*

Lion: *Panthera leo*

Zebra: *Equus zebra*

Kudu: *Tragelaphus strepsiceros*

Bushbaby: *Galago senegalensis*

Sable antelope: *Hippotragus niger*

Ovine: *Ovus spp.*

Monkey: Unknown species

Rodent: Unknown species

Bat: Unknown species
