# Supplementary material for: Epidemiological aspects of the persistent transmission of rabies during an outbreak (2010 – 2017) in Harare, Zimbabwe
Source: PLoS One. 2019 Jan 10;14(1):e0210018. doi: 10.1371/journal.pone.0210018 (PMC6328171; doi:10.1371/journal.pone.0210018)
Supplement: S3 Table — (PDF) [file pone.0210018.s003.pdf]

S3 Table. Distribution of rabies-positive samples within the suburbs and peri-urban areas of the Harare Metropolitan province during the outbreak, 2010 – 2017.

Suburb	Region	Total number of rabies-positive cases detected in the suburb	Years in which rabies-positive cases were detected in the suburb
Borrowdale	North Eastern region	48	2010, 2011, 2012, 2013, 2014, 2015, 2016, 2017
Hatfield	Southern region	43	2011, 2012, 2013, 2014, 2015, 2016, 2017
Chisipite	North Eastern region	30	2010, 2011, 2012, 2013, 2014, 2015, 2016, 2017
Mount Pleasant	Northern region	19	2011, 2012, 2013, 2014, 2015, 2016, 2017
Greendale	North Eastern region	18	2010, 2011, 2012, 2013, 2014, 2016, 2017
Greystone Park	North Eastern region	10	2011, 2012, 2014, 2015, 2016, 2017
Mandara	North Eastern region	7	2011, 2012, 2014, 2015, 2016
Belvedere	Western region	12	2013, 2014, 2016, 2017
Glen Lorne	North Eastern region	7	2010, 2012, 2016, 2017
Kamfinsa	North Eastern region	6	2011, 2012, 2013, 2016
Tynwald	Western region	7	2011, 2013, 2014, 2015, 2016, 2017
Kuwadzana	Western region	5	2011, 2012, 2013, 2016
Marlborough	Western region	6	2011, 2012, 2014, 2015, 2017
Waterfalls	Southern region	4	2011, 2015, 2016
Westgate	Western region	4	2014, 2016
Avondale	Northern region	5	2014, 2016, 2017
Highlands	North Eastern region	3	2011, 2013, 2016
Msasa Park	Southern region	3	2015, 2016
Cranborne	Southern region	2	2014
Hatcliffe	North Eastern region	2	2012, 2015
Highfield	Southern region	2	2011, 2012
Mabelreign	Western region	3	2011, 2012, 2017
Msasa	Eastern region	2	2016

Strathaven	Western region	2	2012, 2016
Umwinsdale	North Eastern region	2	2014, 2016
Vainona	Northern region	2	2012
Bluff Hill	Western region	2	2017
Alexandra Park	Northern region	1	2011
Ballatyne Park	North Eastern region	1	2012
Bloomingdale	Western region	1	2015
Braeside	Southern region	1	2015
Chadcombe	Southern region	1	2015
Dzivaresekwa	Western region	1	2016
Gun Hill	Northern region	1	2013
Helensvale	North Eastern region	1	2015
Hillside	Eastern region	1	2011
Mbare	Southern region	1	2016
Meyrick park	Western region	1	2013
Millton park	Western region	1	2016
New Marlbrough	Western region	1	2016
New Prospect	Southern region	1	2015
Sunningdale	Southern region	1	2013
Tafara	Eastern region	1	2016
The Grange	North Eastern region	1	2012
Tynwald South	Western region	1	2012
Willowvale	Southern region	1	2016
Queensdale	Southern region	1	2016
Belgravia	Northern region	1	2017
Budiriro	Southern region	1	2017
Prospect	Southern region	1	2017
Unknown Suburb	Unknown region	13	2011, 2013, 2016, 2017

Chitungwiza*	Chitungwiza	10	2013, 2015, 2016
Ruwa*	Ruwa	8	2011, 2012, 2013, 2014, 2016, 2017
Epworth*	Epworth	5	2011, 2015, 2016
Goromonzi*	Goromonzi	1	2010
* Cities and/or large metropolitan towns outside the Harare city limits			
